# Intensive statin treatment ameliorate the Th17/Treg functional imbalance in patients with non‐ST elevation acute coronary syndrome underwent percutaneous coronary intervention

**DOI:** 10.1002/clc.23326

**Published:** 2019-12-24

**Authors:** Xiaojing Ma, Shilei Liu, Teng Li, Haitao Yuan

**Affiliations:** ^1^ Department of Cardiology Shandong Provincial Hospital Affiliated to Shandong University Jinan P.R. China

**Keywords:** acute coronary syndrome, inflammation, statin, Th17/Treg

## Abstract

**Background:**

Inflammation plays important roles in the pathogenesis of acute coronary syndrome (ACS). Statins exert positive effects on the plaque stabilization through anti‐inflammation, however, the detailed mechanism is still under investigation.

**Hypothesis:**

Studies suggest that the Th17/Treg functional imbalance takes key part in the plaque destabilization and the onset of ACS. We hypothesized that intensive statin therapy could ameliorate the Th17/Treg imbalance in patients with ACS.

**Methods:**

Sixty‐six patients with non‐ST elevation acute coronary syndrome (NSTE‐ACS) were randomized to conventional group and intensive group. Peripheral blood samples were collected on admission and after atorvastatin treatment. The frequencies of circulating Th17 cells and Treg cells, the levels of cytokines associated with Th17 cells (IL‐17, IL‐6 and IL‐23) and associated with Treg cells (IL‐10 and TGF‐β1) were measured through flow cytometry and ELISA assay respectively.

**Results:**

One week after therapy, the frequencies of circulating Th17 cells of both the groups decreased and the frequencies of circulating Treg cells increased significantly, compared with the basal levels. Furthermore, the decreased frequencies of circulating Th17 cells and the increased frequencies of circulating Treg cells in the intensive group were significantly higher than those in the conventional group. In consistence, the decreased accumulation of IL‐17, IL‐6 and IL‐23 (cytokines relevant to Th17 cells) and the increased accumulation of IL‐10 and TGF‐β1 in peripheral blood were displayed in both groups. The changes are more significant in the intensive group.

**Conclusion:**

Intensive statins therapy could ameliorate the Th17 and Treg functional imbalance in patients with ACS.

## 1 INTRODUCTION

Non‐ST elevation acute coronary syndrome (NSTE‐ACS, including unstable angina pectoris and non‐ST elevation acute myocardial infarction) occurs as a consequence of coronary plaque erosion or rupture. It is thought that inflammation and immune mechanisms play an important role in the pathogenesis of ACS. Recently, CANTOS study^1^ showed that pure anti‐inflammatory treatment could significantly reduce cardiovascular events, which provided definitive evidence‐based medical evidence for the atherosclerotic inflammation hypothesis. Th17 and Treg are two subpopulations of CD4+ T lymphocytes and the Th17/Treg functional imbalance may be important in the pathogenesis of plaque destabilization and the onset of ACS.^2^, ^3^ Early and intensive statin treatment is an efficient intervention to reduce hard cardiovascular events after ACS.^4^ Studies showed that statins have immunomodulatory property and the effect of statins on atherosclerosis depends in part on the immunomodulatory mechanism.^5^, ^6^ However, the mechanisms of Th17/Treg imbalance in ACS patients and the effect of statins on it are still unclear. Our study observed the effect of intensive statin therapy on the Th17/Treg functional imbalance in patients with ACS.

## 2 PATIENTS AND METHODS

### 2.1 Study patients

From January 2017 to December 2018, 72 patients (43 men, 23 women, mean age 66.42 ± 9.68 year) were recruited in the study. Patients were included if they had: (a) diagnosed as NSTE‐ACS; (b) prepared for selective PCI. The exclusion criteria: sever renal and hepatic dysfunction, immunological or infectious disease, tumor, thyroid diseases, immunosuppressive therapy within 1 month. In addition, 25 patients with stable angina were observed as a control group (17 males and 8 females), aged 42‐84 years (mean age 64.48 ± 11.91 years). This study was approved by the ethics committee for clinical investigations of Shandong Provincial Hospital affiliated to Shandong University and informed consent was obtained from the patient's family.

### 2.2 Treatments

All patients were treated with dual antiplatelet therapy consisting of aspirin 100 mg once a day and clopidogrel 75 mg once a day or ticagrelor 90 mg twice a day. β‐Blocker, angiotensin‐converting enzyme inhibitor/angiotensin receptor blocker, nitrates and low molecular weight heparin were administered by the attending physician when necessary. Patients were randomized to two groups, the conventional group with 20 mg atorvastatin and intensive group with 40 mg atorvastatin(Pfizer Ireland Pharmaceuticals，SFDA: J20120049). All the patients underwent selective PCI during hospital.

### 2.3 Laboratory investigations

Blood samples were withdrawn on admission for measuring low‐density lipoprotein cholesterol (LDL‐C), serum creatinine (Scr), high‐sensitivity troponin T (hsTnT), and N‐terminal pro‐brain natriuretic peptide (NT‐proBNP) with automatic biochemistry analyzer.

On admission and 1 week after statin therapy, blood samples were withdrawn for cytometric analysis of the stained Th17 cells (counted as CD4 + IL‐17A + Th17/CD4 + T cells) and Treg cells (counted as CD4 + CD25 + Foxp3Treg/CD4 + Treg cells) by the standard flow cytometry technique (BD Corporation, USA). Human peripheral blood mononuclear cells (PBMC) were isolated from whole peripheral blood by Ficoll density gradient centrifugation and were suspended at a density of 10^7^cells/ml in complete culture medium. The cell suspension was stimulated with Cell Stimulation Cocktail plus protein transport inhibitors (Biosciences) and put into the incubator. After 4‐6 hours of culture, the cell suspension were transferred to the flow tubes and incubated with Fluorescein isothiocyanate (FITC) anti‐human CD4 (eBioscience) at 2‐8°C for 30 min. Then IC Fixation Buffer (Biosciences) and phyocoerthrin (PE) antihuman IL‐17A (eBioscience) were put into the flow tubers for Th17 analysis. As for Treg analysis, cell suspension was incubated with FITC antihuman CD4 (eBioscience), and allophycocyanin antihuman CD25 (eBioscience) at 2‐8°C for 30 min. Afterwards Foxp3 Fixation/Permeabilization (eBioscience) and PE antibody‐FOXP3 (eBioscience) were put into the flow tubers. Stained cells were analyzed by flow cytometric analysis using flow cytometry (BD Corporation).

The levels of IL‐17, IL‐10, IL‐6, and IL‐23 in plasma were measured by Milliplex multi‐factor assay kit (Luminex, Merck, Germany), following the manufacturer's instructions. The level of TGF‐β1 were measured by enzyme‐linked immunosorbent assay (ELISA), following the manufacturer's instruction (TGF‐β1 ELISA kits from MultiSciences Corporation, China).

### 2.4 Statistical analysis

All statistical analyses were performed using SPSS 20.0 (SPSS Inc., Chicago, Illinois,). *P* < .05 was considered to indicate statistical significance. Continuous variables are presented as mean ± SD, differences between groups were compared by the method of one‐way ANOVA. Categorical variables are presented as number and percentage, and the distribution of these variables between two groups was compared using Chi‐squared test.

## 3 RESULTS

### 3.1 Patient characteristics

Baseline characteristics of the two groups were listed in Table 1. There were no significant differences between the two groups with regard to age, sex, mean blood pressure, heart rate, medications, number of stents, and doses of contrast. There were no major complications in either group and all survived to hospital discharge. The levels of hsTnT, NT‐proBNP, LDL‐C, Scr had no significant difference in both of the two groups on admission.

**Table 1:** Baseline characteristics

Parameters	Conventional group (n = 32)	Intensive group (n = 34)
Age (year)	67.13 ± 9.44	65.76 ± 10.00
Gender (male/female)	20/12	23/11
Mean blood pressure (mm Hg)	75.34 ± 16.21	77.32 ± 14.16
Heart rate (beat/min)	86.94 ± 25.90	82.62 ± 25.71
Hypertension (%)	13(40)	15(44)
Diabetes (%)	9(28)	11(32)
Smoking (%)	8(25)	10(29)
Statins (%)	9(28)	11(32)
Angiotensin‐converting enzyme inhibitor/angiotensin receptor blocker (%)	9(28)	11(32)
β‐Blockers (%)	10(31)	12(35)
Number of stents	1.72 ± 0.59	1.82 ± 0.57
Doses of contrast	151.41 ± 48.73	157.06 ± 46.30
High‐sensitivity troponin T (pg/mL)	297.19 ± 396.58	382.83 ± 406.58
N‐terminal pro‐brain natriuretic peptide (pg/mL)	954.96 ± 1332.63	1086.21 ± 1337.35
Low‐density lipoprotein cholesterol (mmol/L)	2.71 ± 0.85	2.94 ± 0.92
Scr (μmol/L)	70.63 ± 19.40	70.39 ± 19.73

### 3.2 Circulating Th17 cells frequencies

**Figure 1:**
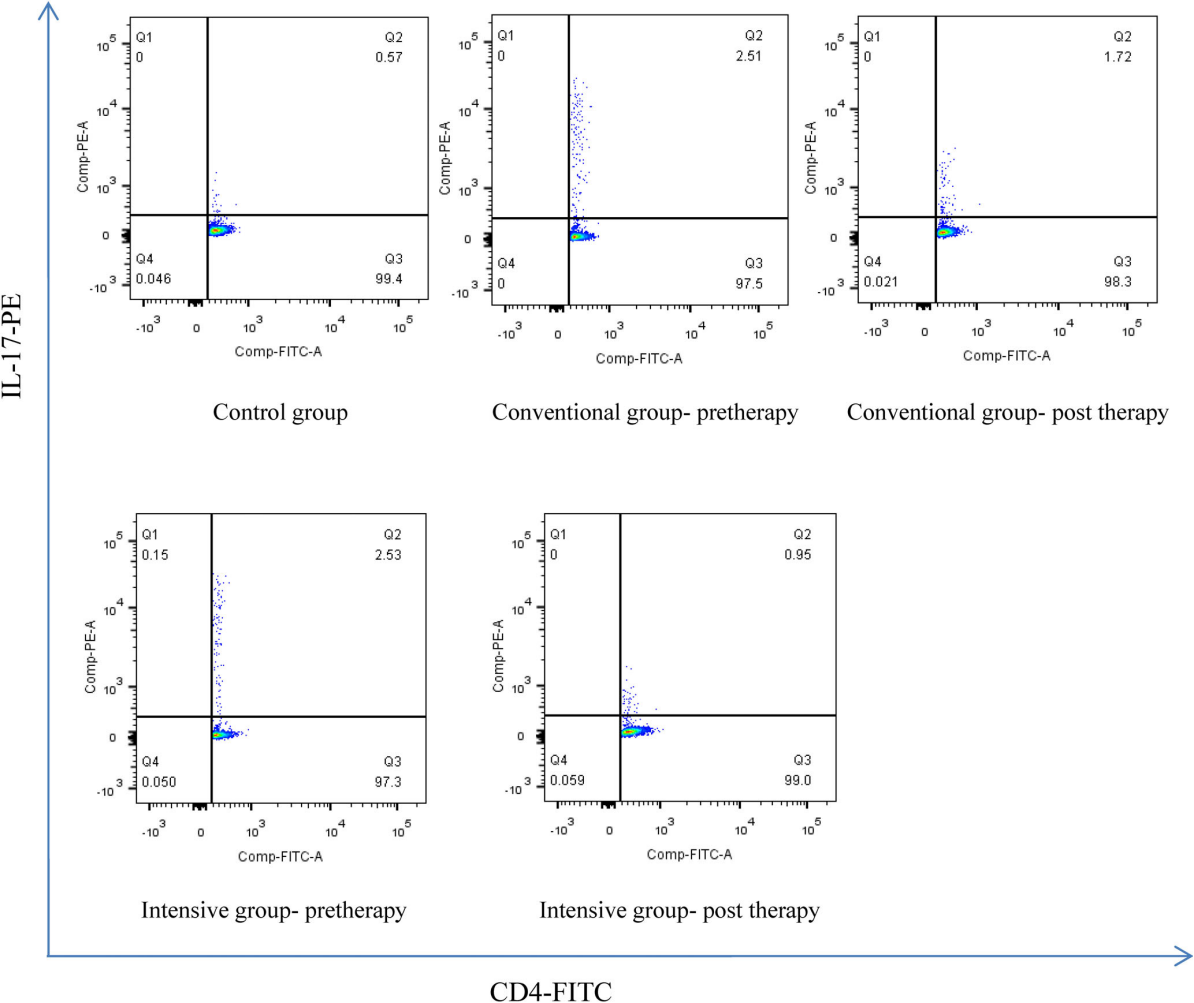
The frequencies of circulating Th17 cells in the conventional group and intensive group were significantly higher than that in the control group on admission (*P* < .05). One week after statins therapy, the frequencies of circulating Th17 cells of both the groups decreased significantly. The decreased frequencies of circulating Th17 cells in the intensive group were significantly higher than that in the conventional group

The circulating Th17 cells frequencies in patients of the control group was 0.63 ± 0.25%. The circulating Th17 cells frequencies in patients with ACS were listed in Table 2 and Figure 1. The frequencies of circulating Th17 cells in the conventional group and intensive group were significantly higher than that in the control group on admission (*P* < .05). There was no significant difference between the two groups. One week after statins therapy, the frequencies of circulating Th17 cells of both the groups decreased significantly. Furthermore, the decreased frequencies of circulating Th17 cells in the intensive group were significantly higher than that in the conventional group.

**Table 2:** Circulating Th17 and Treg cells frequencies in patients

	Th17		Treg	
	Conventional group Intensive group		Conventional group Intensive group	
Pretherapy (%)	2.32 ± 0.64	2.46 ± 0.89	1.48 ± 0.70	1.51 ± 0.76
After therapy (%)	1.50 ± 0.70*	0.86 ± 0.41*^▲^	2.44 ± 1.23*	3.17 ± 1.50*^▲^
Δcells (%)	0.82 ± 0.31	1.59 ± 0.54^▲^	0.95 ± 0.57	1.66 ± 0.71^▲^

*Note*: Compared with pretherapy *P<.05; Compared with conventional group ^▲^ *P*<.05.

### 3.3 Circulating Treg cells frequencies in patients with ACS

**Figure 2:**
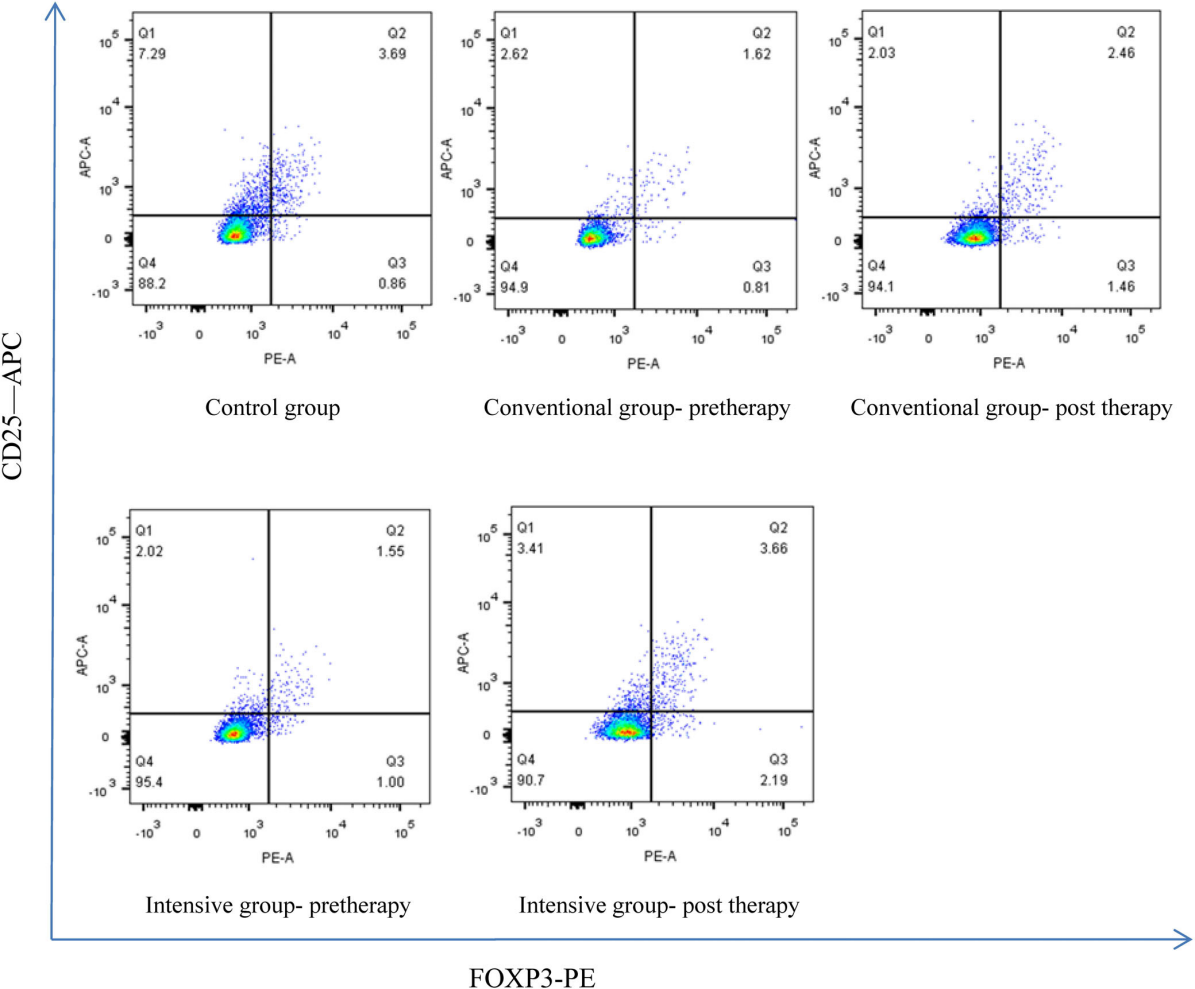
The frequencies of circulating Treg cells in the conventional group and intensive group were significantly lower than that in the control group on admission (*P* < .05). One week after statins therapy, the frequencies of circulating Treg cells of both the groups increased significantly. The increased frequencies of circulating Treg cells in the intensive group were significantly higher than that in the conventional group

The circulating Treg cells frequencies in patients of the control was 3.81 ± 1.52%. The circulating Treg cell frequencies in patients with NSTE‐ACS were listed in Table 2 and Figure 2. The frequencies of circulating Treg cells in the conventional group and intensive group were significantly lower than that in the control group on admission (*P* < .05). There was no significant difference between the two groups. One week after statins therapy, the frequencies of circulating Treg cells of both the groups increased significantly. Furthermore, the increased frequencies of circulating Treg cells in the intensive group were significantly higher than that in the conventional group.

### 3.4 Levels of the important cytokines associated with Th17 and Treg

The levels of important cytokines associated with Th17 cells (IL‐17, IL‐6, and IL‐23) in patients of the control group were 21.98 ± 12.49, 13.53 ± 6.62, and 151.62 ± 58.70 pg/mL. The levels of important cytokines associated with Th17 cells (IL‐10 and TGF‐β1) were 13.44 ± 6.58 and 808.58 ± 410.21 pg/mL. The levels of IL‐17, IL‐6, and IL‐23 in the conventional group and intensive group were significantly higher than those in the control group on admission (*P* < 0.05), while the levels of IL‐10 and TGF‐β1 were significantly lower than those in the control group (*P* < .05). There were no significant differences between the two groups. One week after statins therapy, the levels of IL‐17, IL‐6, and IL‐23 in both the conventional group and the intensive group decreased, and which decreased much higher in the intensive group than the conventional group. While, the levels of IL‐10 and TGF‐β1 in both the groups increased and which increased much significantly in the intensive group than the conventional group (Table 3).

**Table 3:** The levels of IL‐17, IL‐6, IL‐23, IL‐10, and TGF‐β1

	Conventional group (n = 32)	Intensive group (n = 34)
IL‐17 (pg/mL)		
Pretherapy	70.19 ± 28.13	71.50 ± 31.55
Post‐therapy	41.54 ± 17.43*	32.20 ± 12.90*^▲^
ΔIL‐17	28.64 ± 14.21	39.29 ± 19.95^▲^
IL‐6 (pg/mL)		
Pretherapy	49.19 ± 22.08	48.28 ± 20.80
Post‐therapy	32.98 ± 15.99*	16.45 ± 8.16*^▲^
△IL‐6	16.20 ± 8.87	31.82 ± 13.67^▲^
IL‐23 (pg/mL)		
Pretherapy	669.25 ± 311.01	31.82 ± 13.67^▲^
Post‐therapy	296.78 ± 175.08*	684.27 ± 355.42
△IL‐23	372.47 ± 171.80	202.68 ± 107.94*^▲^
IL‐10 (pg/mL)		
Pretherapy	6.06 ± 2.71	6.20 ± 3.18
Post‐therapy	9.95 ± 4.55*	12.28 ± 3.94*^▲^
△IL‐10	3.89 ± 2.46	6.08 ± 1.86^▲^
TGF‐β1 (pg/mL)		
Pretherapy	391.90 ± 192.67	398.81 ± 189.42
Post‐therapy	650.14 ± 327.70*	834.31 ± 370.05*^▲^
△TGF‐β1	258.24 ± 173.69	435.50 ± 214.92^▲^

*Note*: Compared with pretherapy *P<.05; Compared with conventional group ^▲^ *P*<.05.

## 4 DISCUSSION

The activation of immunity is closely related to atherosclerosis, and the imbalance of regulatory and pathogenic immunity may promote the development of atherosclerosis.^7^ Atherogenesis involves various immune cells, particularly CD4+ T‐helper cells.^8^, ^9^ Th17 and Treg cells, as newly discovered CD4+ T cells, are closely related to inflammation, which might affect the plaque degeneration and the onset of ACS. Th17 cells are important components of activated T cells, which played a key role in the progression of plaque instability.^10^, ^11^ Th17 cells produce the signature cytokines interleukin‐17 (IL‐17), and to a lesser extent tumor necrosis factor‐α (TNF‐α), IL‐23, and IL‐6. IL‐17 can also stimulate macrophages to produce inflammatory cytokines, such as IL‐6, TNF‐ a, and IL‐1b.^12^, ^13^ Zhu et al^14^ demonstrated that IL‐17 not only promotes the production of large amounts of vWF by vascular endothelial cells, but also induces apoptosis of vascular endothelial cells through the mitochondrial pathway. IL‐6 is a cytokine that had been implicated in vascular inflammation.^15^ IL‐6 receptor blocker with tocilizumab could reduce the expression level of C reactive protein and troponin in patients with non‐ST segment elevation myocardial infarction.^16^ Our study showed that the frequencies of circulating Th17 cells and the levels of IL‐17, IL‐6, and IL‐23 in patients with ACS were significantly higher than those in the patients of the control group, which suggested that Th17 cell‐mediated immune response may promote the inflammatory progression of plaque destabilization and the onset of ACS.

Treg cells are a unique subset of T cells, which have an indispensable role in the immune system.^17^ Treg cells mediated immunosuppression and prevented the activation of T cells. It had been suggested that Tregs might have an important protective role in the development and progression of atherosclerosis.^18^ Studies showed that Treg cells have an anti‐inflammatory role and maintain the homeostasis of cell subsets involved in the adaptive immunity by contact‐dependent suppression or by releasing anti‐inflammatory cytokines, such as IL‐10 and TGF‐β1.^19^ Active TGF‐β1 is important for Tregs to mediate immunosuppression.^20^ IL‐10 is an immunoregulatory cytokine and had been shown to have a protective role in both atherosclerotic lesion formation and stability in animal studies.^21^ In our study, the frequencies of circulating Treg cells and the levels of TGF‐β1 and IL‐10 in the conventional group and intensive group were significantly lower than those in the control group on admission.

Statins could reduce the risk of cardiovascular diseases and had been widely used in the prevention and therapy of coronary heart disease. Statins also had anti‐inflammatory and immunomodulatory effects. A number of recent studies have shown that statins could improve the imbalance of Th17/Treg cell function in many immune system diseases, such as Kawasaki disease, asthma, and hypertension. Meng et al^22^ had shown that statins therapy improves the quantity and suppressive function of Treg cells in patients with ACS. But, there is no clinical evidence that intensive statins therapy is beneficial to improve the imbalance of Th17/Treg cells in patients with ACS. In our study, patients with NSTE‐ACS were enrolled and were given conventional statin therapy and intensive statin therapy separately. Results showed that: (a) 1 week after atorvastatin therapy, the circulating Th17 cells frequencies and the levels of IL‐17, IL‐6, IL‐23 in patients with NSTE‐ACS decreased. The decreased frequencies and levels in the intensive group were significantly higher than those in the conventional group; (b) 1 week after statins therapy, the frequencies of circulating Treg cells and the levels of TGF‐β1 and IL‐10 of both the groups increased significantly. The increased frequencies and levels in the intensive group were significantly higher than those in the conventional group.

The results suggested that the intensive statins therapy is more conducive to exerting anti‐inflammatory effects by inhibiting Th17 cell‐mediated pathogenic immune response.

### 4.1 Limitations

Our study has some limitations. First, guidelines recommended early initiation of statin therapy for patients with ACS. Therefore, based on ethical considerations, we could not set patients who were not treated with statins as a control group. Second, the present cohort consisted of a small number of patients and shorter observation time. The present results therefore require verification in a larger population and longer follow up.

## SOURCES OF FUNDING

This work was supported by the National Natural Science Foundation of China (No.81770382).The funders had no role in study design, data collection and analysis, decision to publish, or preparation of the manuscript. The authors received no specific funding for this work.

## CONFLICT OF INTEREST

The authors declare no potential conflict of interests.
